# Classic Hodgkin's Lymphoma With Epstein–Barr Viremia and Lymphadenopathy

**DOI:** 10.1155/carm/4599207

**Published:** 2025-07-29

**Authors:** Zulma D. Sosa Carcamo, Salvador Alvarez, Joan M. Irizarry Alvarado

**Affiliations:** Division of General Internal Medicine, Mayo Clinic, 4500 San Pablo Rd S, Jacksonville 32224, Florida, USA

**Keywords:** Epstein–Barr virus infection, Hodgkin's lymphoma, lymphadenopathy, Reed–Sternberg cell

## Abstract

Hodgkin's lymphoma (HL) is uncommon, and its etiology has been attributed to infectious sources such as Epstein–Barr virus (EBV). Though pathogenesis is not completely understood, studies have revealed that specific viral proteins from EBV conduct the process of HL development. In this report, we will discuss the case of a patient who developed EBV-associated classic HL 15 years after an episode of infectious mononucleosis.

## 1. Introduction

Epstein–Barr virus (EBV) was recognized for the first time in cancer cells of Burkitt's lymphoma [1]. Later, it was found in other malignancies such as nasopharyngeal carcinoma, non-Hodgkin's lymphoma (HL) and HL, gastric carcinoma in immunocompetent patients, and lymphoproliferative diseases in immunocompromised patients, revealing its connection to malignancies. EBV is a DNA virus and belongs to the group of herpes viruses [1–3]. It is transmitted primarily through saliva, but it can also be disseminated through breast milk, and by an infected solid organ transplantation [2]. EBV initially infects epithelial cells in the oropharynx where it replicates, but its primary target is B cells, where it remains latent after primary infection [1, 2].

This is the case of a patient diagnosed with classical HL (cHL) with suspected reactivation of latent EBV, who presented with significant lymphadenopathy and EBV viremia.

## 2. Case Presentation

A previously healthy 38-year-old Hispanic female presented with painless right neck mass associated with upper respiratory infection that appeared approximately 2 weeks prior to our evaluation. Local evaluation with a CT scan of the neck and chest confirmed the presence of multiple enlarged lymph nodes on both sides of the neck and the mediastinum. She continued her daily activities and exercise routine. She denied fever, chills, rash, pruritus, night sweats, or weight loss. Also, she denied animal contact, except for her cat, which she has minimal contact with. The patient gave a history of infectious mononucleosis (IM) in the past. The patient lives in Honduras and had recently traveled to the Bahamas on a cruise. She denied any risk factors for HIV or tuberculosis. The patient consumes uncooked meat in the form of carpaccio.

**Figure 1:**
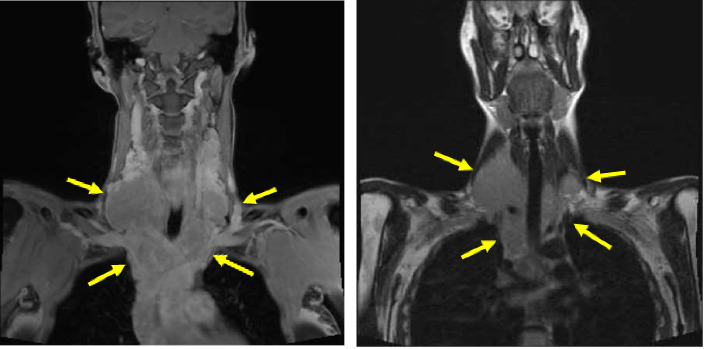
MRI of the neck shows bilateral (right greater than left) cervical and mediastinal lymphadenopathy (yellow arrows).

**Figure 2:**
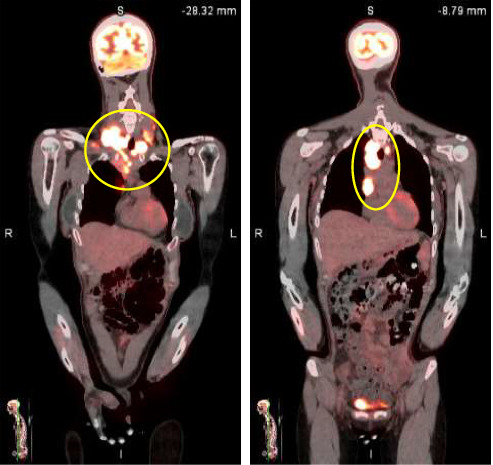
PET CT shows hypermetabolic cervical and mediastinal lymphadenopathy (yellow circles).

Neck MRI showed bilateral supraclavicular and subcarinal lymphadenopathy. CT of the abdomen/pelvis showed diffuse lymphadenopathy. PET scan showed multiple marked hypermetabolic lymphadenopathy (see Figures 1 and 2).

Laboratories revealed mild anemia and multiple negative infectious disease serologies. EBV studies were consistent with a past infection (negative IgM for VCA, positive IgG for VCA, and positive EBNA IgG). EBV DNA polymerase chain reaction (PCR) detected and quantified 2020 IU/mL.

Ultrasound-guided biopsy of the neck was obtained revealing large, atypical cells that were positive for CD15, CD30, PAX5, and MUM1 with variable expression of CD20. They were negative for CD19, CD79a, BCL6, and CD45. CD3 is positive for a fair amount of T lymphocytes, which are positive for CD5 with mixed CD4 and CD8 expression. Keratin, melan A, and S100 are negative. In situ hybridization studies for EBV are positive in the large, atypical cells. Molecular studies by PCR are positive for a clonal B cell gene rearrangement and negative for T cell receptor gene rearrangement. These results were consistent with cHL (EBV positive). Bone marrow biopsy was not obtained. Based on clinical presentation and imaging, the patient was classified as Ann Arbor Stage III.

She was referred to oncology, and after reviewing the available imaging and pathology, they decided to start the patient on nivolumab, doxorubicin, vinblastine, and dacarbazine. After the first cycle, lymphadenopathy shrank dramatically, and the patient responded very well to treatment. EBV DNA levels after three cycles of chemotherapy were undetectable.

## 3. Discussion

More than 90% of the global population is infected with EBV [1–4]. In developing countries, poor socioeconomic conditions such as overcrowding and inadequate sanitation can facilitate early exposure to EBV [3, 5, 6]. Primary EBV infection in young children is typically asymptomatic or resembles other mild viral syndromes. In contrast, primary infection during adolescence or adulthood results in IM. IM is characterized by pharyngitis/tonsillitis, fever, malaise, lymphadenopathy, and atypical lymphocytosis. In severe cases, patients can develop hepatomegaly and splenomegaly [1, 3, 7, 8]. While 30%–50% of individuals who experienced primary infection with EBV develop IM or mild acute infection, the other 50% infected remain asymptomatic [1]. EBV expresses different viral genes and proteins that promote proliferation, differentiation, and transformation of infected B cells, which consequently produce clonal B cells containing the viral genome. Downregulation of some EBV viral proteins in these cells allows the virus to evade immune detection, allowing the viral DNA to remain inside the nucleus of the host B cell. This is known as latency [1–4, 9, 10].

HL is uncommon, with approximately 9000 annual cases in the United States. The subtype associated with EBV is cHL. Although the pathogenesis behind this association is unclear, latent EBV genomes have been identified in Reed–Sternberg cells in 30%–50% of all HL cases (EBV-positive HL) [2–4, 6]. In developed countries, HL incidence peaks among young adults aged 15–35 years, whereas in developing countries, the highest rates are seen in children under 10 years and adults over 45 years of age [4, 5].

This case is of particular interest given its unique clinical manifestation and the timeline of presentation. Our patient presented to the clinic after 2 weeks of upper respiratory symptoms and lymphadenopathy with a notable history of IM 15 years prior. Biopsies of lymph nodes confirmed EBV-positive cHL, and blood work demonstrated past EBV exposure with an elevated viral load. Although this should not be considered causal, we can presume that viral reactivation may have taken place. While the pathogenesis linking EBV and HL remains unclear, studies have reported a median interval of 2.5–4 years between IM and the development of EBV-positive HL [5–7, 9]. In contrast, our patient developed HL 15 years after the initial episode of IM, making this notably uncommon with a delayed presentation.

Spacek et al. reported that high plasma EBV DNA levels can be associated with the presence of HL and can be used as a monitor of chemotherapy response. In their study, free plasma EBV DNA levels declined after chemotherapy, supporting its potential utility in tracking disease activity. Notably, in one case, EBV DNA levels became detectable in plasma after 2 years of chemotherapy even prior to clinical/radiographic evidence of relapse. This suggests that EBV viral load can be useful in the prognosis and early detection of recurrence [6]. The discovery of the relation between EBV DNA plasma levels and HL has contributed to identifying patients at risk of EBV-associated diseases and was suggested as an instrument to monitor these malignancies [3, 5, 10]. In addition, EBV DNA has been identified in the majority of EBV-positive HL patients and typically becomes undetectable after chemotherapy [8, 11]. In our patient, EBV DNA levels became undetectable after three cycles of chemotherapy, supporting these observations. However, it remains unclear whether the elevated EBV DNA levels are caused by a reactivation of EBV DNA replication, or these only reflect a marker of viral DNA released from tumor cells.

The most used combinations of chemotherapy in treating HL are doxorubicin, bleomycin, vinblastine, and dacarbazine (ABVD), and bleomycin, etoposide, doxorubicin, cyclophosphamide, vincristine, procarbazine, and prednisone (BEACOPP). A recent clinical trial evaluating the addition of nivolumab to ABVD regimen showed a 2-year progression-free survival rate of 92%, compared to 83% in the brentuximab arm [12]. Based on this study, the patient and oncologist decided to proceed with the ABVD plus nivolumab regimen.

## 4. Conclusion

In summary, PCR has facilitated the detection of EBV DNA in humans, allowing identification of the virus in patients and its association with malignancies. Half of all annual HL cases are linked to EBV. These findings underscore the need for further research to prevent virus-associated malignancies.

## Data Availability

Data sharing is not applicable to this article as no new data were created or analyzed in this study.
